# Adapting Tai Chi for Upper Limb Rehabilitation Post Stroke: A Feasibility Study

**DOI:** 10.3390/medicines4040072

**Published:** 2017-09-30

**Authors:** Shujuan Pan, Dahlia Kairy, Hélène Corriveau, Michel Tousignant

**Affiliations:** 1 School of Rehabilitation, Université de Montréal, Montréal, QC H3N 1X7, Canada; shujuan.pan@umontreal.ca; 2 Centre for Interdisciplinary Research in Rehabilitation of Greater Montreal—IRGLM Site, Montreal, QC H3S 2J4, Canada; 3 School of Rehabilitation, Université de Sherbrooke, Sherbrooke, QC J1H 5N4, Canada; Helene.Corriveau@USherbrooke.ca (H.C.); Michel.Tousignant@USherbrooke.ca (M.T.); 4 Research Center on Aging, Centre intégré universitaire de santé et des services sociaux de l’Estrie-CHUS, Sherbrooke, QC J1H 4C4, Canada

**Keywords:** stroke, rehabilitation, upper extremity, tai chi, feasibility

## Abstract

**Background:** Tai chi (TC) has been reported as being beneficial for improving balance post stroke, yet its utility in upper limb rehabilitation remains unknown. **Methods:** Twelve chronic stroke survivors with persistent paresis of an upper limb underwent 60 minutes of adapted TC twice a week for eight weeks, with a 4-week follow up. A 10-min TC home program was recommended for the days without sessions. TC level of performance, attendance to the sessions, duration of self-practice at home, and adapted TC movements used were recorded. **Results:** Eleven participants completed the study. A clinical reasoning algorithm underlying the adaptation of TC was elaborated throughout the trial. Participants with varying profiles including a severely impaired upper limb, poor balance, shoulder pain, and severe spasticity were not only capable of practicing the adapted TC, but attended all 16 sessions and practiced TC at home for a total of 16.51 ± 9.21 h. The degree of self-practice for subgroups with low upper limb function, shoulder pain, or moderate-to-severe spasticity was similar to that of subgroups with greater upper limb function, no shoulder pain, and minimal-to-no spasticity. **Conclusion:** Adapted TC seems feasible for upper limb rehabilitation post stroke. Although the study was based on a small sample size and requires confirmation, low upper limb function, insufficient balance, spasticity, and shoulder pain do not appear to hinder the practice of TC.

## 1. Introduction

Stroke is a leading cause of serious, long-term disability among middle-aged and older adults worldwide [1]. Functional impairment of an upper limb is reported in approximately 85% of stroke survivors [2]. The effects of current treatments for arm weakness are shown to be suboptimal [3]. Though upper limb recovery has been found to continue even in the chronic stage [4], long-term rehabilitation services are limited for a large proportion of chronic stroke patients after returning home [5,6]. Therefore, novel and effective approaches are needed to provide timely and ongoing upper limb rehabilitation.

Tai chi is an ancient martial art originating from Chinese healing traditions. Typified by slow and gentle movements, tai chi is an “internal” martial art that cultivates the flow and balance of *qi*, which is translated as vital energy [7]. The relaxation of body and mind is a main feature which is said to facilitate the flow of *qi* [8]. Also, tai chi requires well-coordinated sequencing of segments to make the body move as a whole unit to help the flow of *qi* [9]. Thus, tai chi is a moving form of qigong, which is a practice focusing on cultivation, circulation, and harmonization of *qi*. To date, tai chi is accepted as a suitable, low impact, home-based exercise option for aged and patients with chronic diseases [10,11,12]. Since tai chi emphasizes slow and continuous weight transfer between the two feet, it has been widely shown as beneficial for improving balance and for fall prevention in the aged [13,14,15].

In recent years, some studies have also reported the benefits of tai chi in improving balance in chronic stroke patients [16,17,18]. However, the use of tai chi in upper limb rehabilitation post stroke remains unknown. Tai chi is not only an exercise of lower limb, but a whole-body exercise. Upper limb muscle strength and flexibility have been shown to improve in the aged following the practice of tai chi [19,20,21]. Tai chi practitioners have also demonstrated better arm movement control and eye–hand coordination in older adults [21,22,23,24]. Furthermore, the relaxation component of tai chi may have the potential to improve the motor function of the paretic upper limb. Therefore, tai chi may be a promising upper limb rehabilitation method.

However, the presence of hemiplegia may be an important barrier to using tai chi for upper limb rehabilitation post stroke, potentially limiting the ability to actually perform upper limb tai chi movements. Similarly, shoulder pain and severe spasticity of the affected arm may impact on the ability to perform tai chi movements. Furthermore, the standing position used in traditional tai chi styles poses difficulties for persons with poor balance. Adapting tai chi to take into account these limitations may need to be included in post-stroke rehabilitation. Although sitting tai chi has been reported to be used in persons with spinal cord injuries to improve muscle strength of the upper limbs [25], little is known about how to adapt tai chi with respect to paretic upper limbs. Moreover, the feasibility of using adapted tai chi movements for upper limb rehabilitation remains unknown.

Therefore, this study aimed to explore the use of adapted tai chi movements for upper limb rehabilitation. More specifically, the objective was to evaluate the feasibility of using adapted tai chi for upper limb rehabilitation post stroke, including: (1) whether the adapted tai chi was performable and acceptable by participants; and (2) whether the potential influencing factors such as impairment level of an upper limb, insufficient balance, shoulder pain, and spasticity constrained the practice of the adapted tai chi. A second objective was to document the clinical reasoning underlying the adaptations made to tai chi based on the participants’ characteristics when used for upper limb rehabilitation post stroke. The efficacy of adapted tai chi has been reported elsewhere [26].

## 2. Methods

### 2.1. Study Design

A single-group pre–post design was used in this exploratory study. All participants underwent an 8-week tai chi intervention and a 4-week follow up evaluation.

### 2.2. Participants

A convenience sample of 12 community-dwelling chronic stroke survivors was recruited in this study. The inclusion criteria were: (1) history of stroke with a paretic upper limb, at least 6 months before the start of the study; (2) upper limb recovery in stages 2–6 according to the Chedoke–McMaster Arm Impairment Inventory [27], or presence of upper limb dysfunction as reported by participants in stage 7; and (3) ability to understand the instructions to participate in assessments and tai chi interventions. The exclusion criteria were: (1) uncontrolled medical problems; and/or (2) significant hemianopia or aphasia limiting the ability to participate in the study. The study protocol was approved by the institutional review board of the Center for Interdisciplinary Research in Rehabilitation of Greater Montreal (CRIR). Written informed consent was obtained from all participants before the first evaluation.

### 2.3. Tai Chi Intervention

Participants underwent a 60-min tai chi session twice per week for eight weeks. Sessions were delivered individually by a clinician with two years of tai chi experience in a research center that is part of the CRIR. Each session included a 5-min warm-up sequence, a 5-min cool down sequence, and 50 min of adapted tai chi with rest periods if necessary. A 10-min tai chi home program was also proposed for days without sessions and during the follow-up period (i.e., weeks 9–12). Participants were asked to repeat the tai chi movements at home.

The tai chi intervention consisted of eight forms selected from traditional styles. Two forms derived from the Chen style [28], known as Front Cloud Hands and Side Cloud Hands (see a supplementary video of the article [26]) emphasized abduction, flexion and external rotation. The six forms chosen from the Yang style [29] had an emphasis not only on shoulder abduction and flexion, but also on elbow extension, supination of forearm and dorsiflexion of wrist and fingers. Ranging from simple to complex sequences, these are known as Brush Knees and Push, Parting Wild Horse’s Mane, Fair Lady Works at Shuttles, Parry Block and Punch, Cloud Hands from Yang style, and Step Back and Repulse Monkey [26]. The forms from the Chen style and the first two forms from the Yang style were the basic moves used with all participants; other forms from the Yang style were more difficult and were used depending on the participants’ upper limb function and ability to perform the movements.

The tai chi training followed a gradual, part-to-whole, and easy-to-difficult progression. The instructor selected the appropriate adapted tai chi forms based on each participant’s ability during the sessions. Two key principles were used when adapting the tai chi training for each participant: (1) the participant should be able to practice tai chi movements while relaxing; and (2) there should be as much whole-body coordination as possible. Participants were specifically asked to relax muscle and joints and focus on movements to help relaxation. They were asked to perform the movements with minimal perceived exertion. Movements were practiced slowly, repeatedly and even segmentally if necessary. For participants with low upper limb function, only shoulder and elbow movements were emphasized. Active movements were performed with the affected limb even if the range of motion was small, although assistance using the unaffected hand was allowed at the beginning.

### 2.4. Outcome Measures

The demographic and clinical characteristics of all participants were collected, including age, gender, side of hemiparesis, type of stroke, time since stroke, comorbidities, co-rehabilitation, botulinum toxin injection history, and technical aids for mobility. The Severity Index of Cumulative Illness Rating Scale for Geriatrics (CIRS-G) was used to document their comorbidities with scores ranging from 0 to 4 [30]. The initial arm and hand motor function stages were evaluated using the Chedoke–McMaster Stroke Assessment (CMSA) [27], which has a range of stages from 0 to 7. The Modified Ashworth Scale (MAS) [31,32], a 6-point scale ranging from 0 to 4, was used to grade the spasticity level of paralytic upper limbs.

Session attendance and reasons for dropouts were recorded. Number of times and duration of self-practice at home were recorded by participants in a log book and collected by the instructor at each session and at the end of follow-up period. The instructor adapted tai chi for participants based on initial upper limb function and balance levels of participants. The balance level was divided into two categories: sufficient or insufficient to support standing position to practice tai chi. All decisions made by the instructor regarding the choice of upper limb movements and lower positions during adaptation process, and the adapted tai chi used by participants were documented in a log. The tai chi performance of participants was also documented.

The Visual Analogue Scale (VAS) [33] with scores ranging from 0 to 10 was used to measure shoulder pain of affected arm at baseline, post-treatment and 4-week follow-up. Good concurrent validity, test–retest reliability, and sensitivity to change of the VAS have been reported [34,35,36]. The number of falls during tai chi sessions and self-practice at home was recorded.

### 2.5. Data Analysis

Descriptive statistics were used to analyze all variables. Frequencies were calculated to check extreme values. Participants’ self-practice times were compared between groups of different levels of upper limb function, shoulder pain, and spasticity, though the sample size of subgroups was too small to perform inferential statistical analysis. According to participant characteristics and how tai chi was modified, participants were divided into three types of subgroups for analysis with respect to upper limb function, shoulder pain, and spasticity. These subgroups included: low (stage 2 of the CMSA-arm), middle (stage 3), and high (stage 6 and higher) groups for upper limb function analysis; shoulder pain (VAS > 0) and no shoulder pain (VAS = 0) for the shoulder pain analysis; and moderate to severe (MAS ≥ 2), and slight or no spasticity groups (MAS < 2) for spasticity analysis. Analyses were conducted using the SPSS statistical program version 23.0.

## 3. Results

### 3.1. Participant Characteristics

Twelve chronic stroke survivors participated the study. One withdrew from the study after having participated in three sessions because of lack of public adapted transport services. For the purposes of the exploratory study, the analysis was conducted on the 11 remaining participants. The clinical characteristics of participants are presented in Table 1. They were on average 59.4 ± 13.0 years old and 22.7 ± 17.7 months after stroke onset. Two out of eleven were women, and four had dominant-side hemiparesis. Unexpectedly, three participants participated in other community exercise programs for less than 30 minutes per week. Given that the time spent was much less than that of the tai chi, they were not excluded from the study. Comorbidities included hypertension (*n* = 5), diabetic mellitus (*n* = 2), heart bypass surgery, kidney and pancreas transplantation, bilateral total knee replacement, and extreme obesity (*n* = 1 each).

### 3.2. Adherence to Tai Chi Sessions and Self-Practice at Home

**Figure 1:**
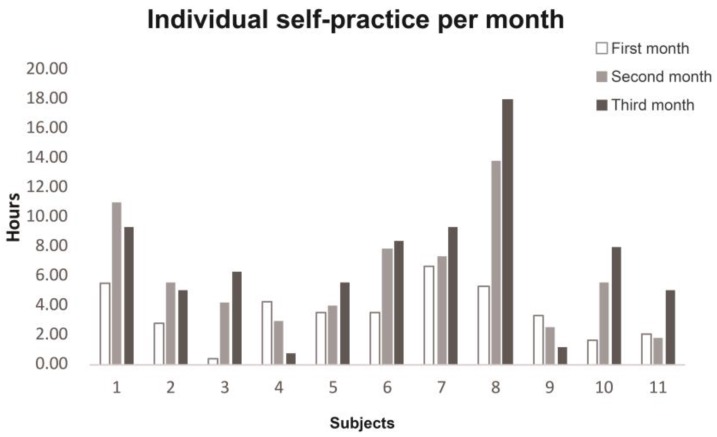
Individual self-practice hours per month of participants.

The 11 participants participated in all 16 sessions of tai chi for two months. After the first session, they were able to begin practicing the adapted tai chi at home. In the first month, 6 participants practiced tai chi at home on more than 17 days, 4 practiced on 9 to 17 days, and 1 participant practiced on 2 days (a total of 0.3 h). However, for this same participant, his self-practice increased to 20 days (4.2 h) and 25 days (6.3 h) in the second and the third months, respectively. In the second and third month, 8 participants practiced more than 17 days, 2 practiced 9 to 17 days, and 1 practiced less than 8 days. They practiced tai chi at home for 5–45 min per day. Their individual self-practice hours per month are presented in Figure 1.

**Figure 2:**
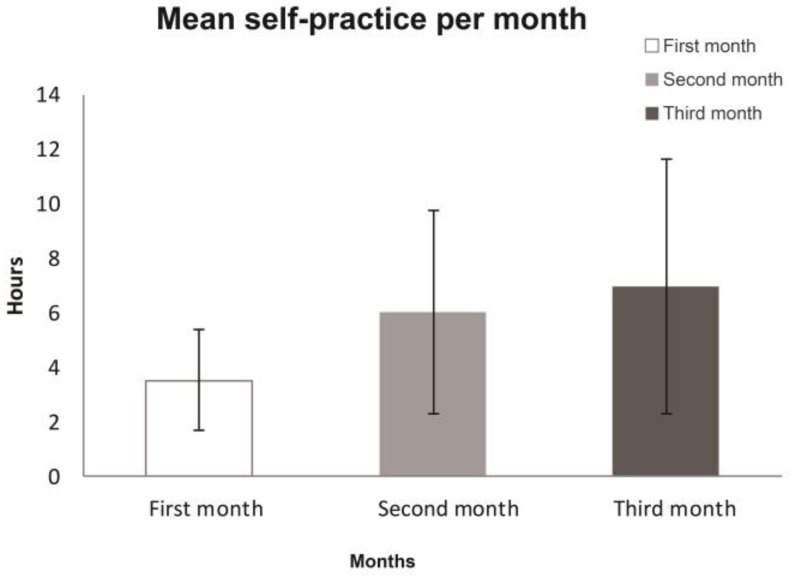
Mean self-practice hours per month of participants.

The mean self-practice time of participants in the first month was 3.5 ± 1.8 h, and this increased significantly in the second and third month by 6.0 ± 3.7 and 6.97 ± 4.7 h, respectively (Figure 2). Only two participants (subjects 4 and 9) decreased their practice in the follow-up by 3 days (0.8 h) and 9 days (1.1 h), respectively, because of lack of motivation without the actual in-person sessions.

### 3.3. Clinical Reasoning Underlying Tai Chi Adaptations

**Figure 3:**
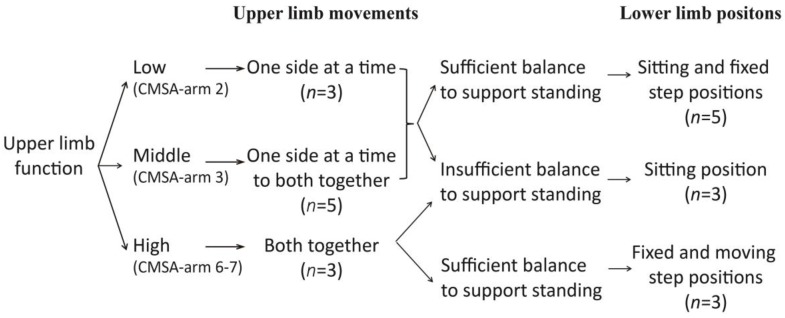
Clinical reasoning for adapting tai chi in the study, including upper limb movements and lower limb positions used by participants. CMSA-arm: arm stage of Chedoke–McMaster Stroke Assessment.

The clinical reasoning process for adapting tai chi during sessions is presented in Figure 3. Participants were asked to practice tai chi with both upper limbs, one side at a time, or both sides together. Their ability to perform upper limb movements was in accordance with the impairment level of the affected upper limb. According to the impairment arm stage of CMSA, participants in stage 2 practiced tai chi with both upper limbs, one side at a time. Only shoulder and elbow movements were emphasized in the affected limb. Participants in stage 3 practiced upper limb movements one side at a time at first and then changed gradually to both sides together after they were familiar with the movements and improved their practice ability. Participants in stage 6 and higher practiced directly both sides together.

Three lower limb positions were employed based on balance level, including sitting, fixed step, and moving step positons. Sitting position was used in participants whose balance was insufficient to support standing. Fixed step and moving step positions were two standing positions used for persons with sufficient balance to support standing. They refer to standing with weight shifting between two feet, with or without actually stepping (moving step position and fixed step position, respectively). Taking upper limb ability into consideration, the moving step position required both high upper limb function and sufficient balance, while the fixed step position could be used in persons with low upper limb function. These three positions were combined in a part-to-whole and easy-to-difficult manner to assist participants to gradually increase their practice ability. Therefore, participants who had sufficient balance to support standing, finally used sitting and fixed step positions when their upper limb function was in stage 2 or 3, while they used fixed step and moving step positions when in stages 6 or 7. Each position occupied half a session.

### 3.4. Tai Chi Performance in Different Situations

#### Upper Limb Impairment Level

**Figure 4:**
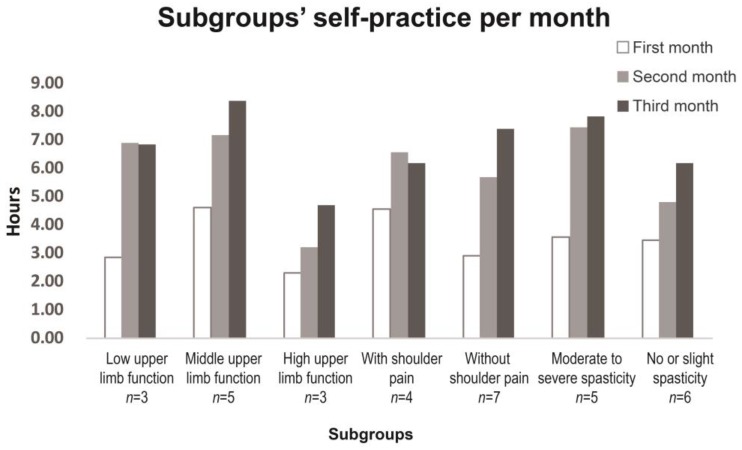
Subgroups’ self-practice hours per month for participants.

According to initial arm stage using CMSA, three participants were in stage 2, five in stage 3, and three in stage 6 and higher (Table 1). Participants in stage 2 had isolated shoulder movements without incorporating the trunk movements before starting the study. They were capable of practicing upper limb movements one side at a time, though motion ranges of affected arms were small when doing movements independently. In addition, they required assistance from the non-affected hand to complete the active movements of the affected limb at the beginning. Five participants in stage 3 practiced both upper limb movements one side at a time first and then changed gradually to both sides once their abilities permitted. Four of them changed to practice both sides together at the end of 16 sessions. Their affected arms could finish movements independently. Participants in stage 6 and higher practiced upper limb movements with sides without modification. The subgroups’ self-practice hours per month are presented in Figure 4. The total self-practice time over the three months (intervention plus follow-up) of the low and middle upper limb functional groups was more than that of the high-functioning upper limb group (16.6 ± 18.1 h, 20.2 ± 11.1 h, and 10.3 ± 4.3 h, respectively).

### 3.5. Insufficient Balance and Falls

Regarding initial mobility aids used, 1 participant used a wheelchair, 4 used a cane, and 6 were independent (Table 1). The participant who used a wheelchair and two participants who used a cane had insufficient balance to support standing and thus practiced tai chi in a sitting position. Three independent participants who were in stage 6 and higher practiced in fixed and moving step positions. Five participants, including 2 using a cane and 3 independent ones, had sufficient balance while they were in stages 2 or 3, and practiced in sitting and fixed step positions. No falls were recorded throughout the study.

### 3.6. Shoulder Pain

Four participants had initial shoulder pain in the affected upper limb before intervention with a mean VAS of 5.5 ± 3, and two of them presented a score over 7 (Table 1). The shoulder pain appeared during movement but did not interfere with tai chi movements. Their shoulder pain decreased with the mean VAS 3 ± 2.8 after intervention and 2.5 ± 2.5 at the end of follow up. Participants without initial shoulder pain did not feel any shoulder pain during the whole study. The total self-practice over the three months was similar in the shoulder pain group (VAS > 0) and no shoulder pain group (VAS = 0) (17.8 ± 7.7 h and 15.4 ± 10.9 h, respectively; Figure 4).

### 3.7. Spasticity

The initial spasticity level of participants is shown in Table 1, with the mean MAS 1.5 ± 1.4. Three participants had severe spasticity (MAS ≥ 3), and required more time to relax before starting a movement as well as segmental pause during movement. All three participants received botulinum toxin injections regularly before participating in the tai chi intervention to reduce spasticity. They did not receive any injections during the study period when reinjection time came. The total self-practice time in three months for the moderate-to-severe spasticity group (MAS ≥ 2) was 18.9 ± 12.2 h, and was 14.5 ± 6.3 h in the slight or no spasticity group (MAS < 2; Figure 4).

## 4. Discussion

The objective of this study was to explore the feasibility of tai chi for upper limb rehabilitation post stoke and its influencing factors. This study suggests that tai chi was feasible for upper limb rehabilitation post stroke after having been adapted to hemiparesis of stroke survivors. Participants with varied characteristics, including a severely impaired upper limb, poor balance, shoulder pain, severe spasticity, high medical comorbidity burden, and the elderly were capable of practicing their selected adapted tai chi movements. Moreover, the adapted tai chi was well accepted by participants. Eleven participants attended all 16 sessions and practiced more than recommended at home even in the follow-up month. The total self-practice hours of subgroups with low upper limb function (stage 2 or 3), shoulder pain (VAS > 0), and moderate and severe spasticity (MAS ≥ 2) were not less than those of their corresponding subgroups, though in this exploratory study sample sizes of subgroups were too small to perform the inferential statistical analysis. Impairment level of the affected upper limb, impaired balance, shoulder pain, and spasticity are not limiting factors for practicing adapted tai chi post stroke. To our knowledge, this is the first study to report the feasibility of tai chi on upper limb rehabilitation after stroke.

A 10-min tai chi home program was recommended for participants on those days without tai chi sessions, in order to help them to continue the following sessions for which difficulty was gradually increasing. This kind of self-administered exercise program by patients during their off-therapy time was recommended and proved effective in improving arm-hand function [37]. Unlike many tai chi programs, the adapted tai chi in the study was simple and easy to learn, so that participants were able to practice it at home after the first session. Their practice at home was in line with expectations in the first month and increased to an extent greater than expected in the second and follow-up month. Possible reasons for this may be that after the first month participants were more familiar with the movements, or they may have been more motivated to practice tai chi at home once they felt some improvement. Interestingly, participants in stages 2 and 3 practiced at home even more than participants in stage 6 and higher. One reason may be that they were more motivated since their upper limb function was relatively low, or perhaps they may have felt more benefits during the study. Another reason may be that they were more available to include tai chi practice into a routine since they were less engaged in daily activities. Future studies should examine the reasons for compliance and non-compliance to better address the barriers.

The underlying clinical reasoning was identified in the study to tailor the tai chi training to different functional levels of the participants. Two main principles were used for adapting tai chi. The first one was to adapt movements of involved upper and lower limbs in order to allow the participants to practice tai chi. For severely impaired upper limbs, several strategies were used to facilitate practice. First, tai chi movements were modified from both sides together to one side at a time. Second, a practice requirement for affected sides was the ability to move mainly the shoulder and elbow, while accuracy was not necessary. Movements were proposed to be practiced slowly and even segmentally if necessary. Moreover, active movements of the affected sides were assisted by the unaffected hands. Consequently, participants in stages 2 and 3 were able to practice their selected upper limb movements, and they practiced tai chi at home with good compliance. Considering that movement practice is dramatically influenced in severely impaired upper limb reported by previous studies [38,39], the capability of participants in stage 2 and 3 to perform multiple repetitions of tai chi movements is already a meaningful advancement. It should be noted that participants in stage 2 in the study were able to have isolated shoulder movements without compensatory the trunk movements before treatment. However, the feasibility of doing tai chi even when adapted with individuals who do not have isolated shoulder movements remains to be tested.

Another principle used when to adapting the tai chi was to provide as much whole body coordination as possible. Coordination is an important feature of tai chi. Moreover, using the principle of coordination can also increase the practice difficulties and challenge participants to improve their ability, which is an important strategy in stroke rehabilitation. Firstly, both upper limbs were required to move together when upper limb function permitted. In contrast to bilateral arm training whereby patients practice the same activities with both upper limbs simultaneously [40], tai chi movements of both upper limbs were different, which is more challenging (i.e., the arm movements performed by each arm were different). From stage 3, participants were encouraged to gradually practice upper limb movements together. Results indicate that four of the five participants progressed to practice with both arms together at the end of 16 sessions, and participants in stage 6 and higher could practice with both arms from the start. Although there were no participants in stages 4 and 5, we tentatively put forward that they may also practice upper limb movements from one side at a time to both together as do participants in stage 3, although this remains to be confirmed in future studies. Previous studies have reported that practicing bilateral movements improved the recovery of the involved upper limb [41]. The activation of the intact hemisphere may facilitate the recovery of the damaged hemisphere through neural networks [42]. Based on original tai chi forms, the bilateral movements in the study were different, which may have a different effect on functional recovery. Further research examining its effects is required.

Regarding the paretic lower limbs, a sitting position was used to replace traditional moving step position by patients without sufficient balance to support standing. Three participants who used a wheelchair or a cane practiced tai chi from a sitting position in the study. Although sitting tai chi has been used in persons with spinal cord injuries to improve muscle strength of upper limb [25], to our knowledge this is the first time it has been reported in stroke rehabilitation. No falls were recorded during the study. Thus, this exploratory study suggests that tai chi can be safe when appropriately adapted to the participants’ balance. However, it should be noted that this study assessed balance level as being able or not to maintain the standing position during the intervention. Future studies should pursue the clinical reasoning in order to include more detailed balance evaluation data.

On the other hand, fixed and moving step positions were used to promote upper and lower limb coordination under the condition of sufficient balance. Considering that sitting position may also help persons with poor upper limb function to better concentrate on upper limb practice, it was also used in the study for those who had sufficient balance but poor upper limb function (stages 2 and 3). Therefore, a combination of lower limb positions was applied. Sitting and fixed step positions were chosen for participants with enough balance while having low upper limb function; fixed and moving step positions used for participants with enough balance and high upper limb function. Furthermore, the combinations took the challenge and ease of exercise into consideration. Since this clinical reasoning included lower limb exercise, it may be used not only for upper limb rehabilitation, but also for rehabilitation of both upper and lower limbs in the future.

Two physiological mechanisms may explain the feasibility of using adapted tai chi for upper limb rehabilitation post stroke. First, the adaptation of tai chi movements to hemiparesis allowed participants to perform multiple repetitions of movements. Even participants with low upper limb function or poor balance were able to perform a high amount of the adapted tai chi. Repetition of active movement practice is thought to correlate largely with motor recovery post stroke [43]. Second, the adapted tai chi was done with relaxation by the participants during the tai chi sessions. Muscle relaxation is the essential feature which differentiates tai chi from many other exercises [44]. Without the need of supporting body weight, upper limbs are thought relax more easily than lower limbs during tai chi practice [45]. Results showed that the three participants with severe spasticity (MAS ≥ 3) were able to follow tai chi sessions given more time for relaxation. They did not receive botulinum toxin reinjections during the study. The total number of self-practice hours of subgroup in MAS ≥ 2 was not less than that of subgroup in MAS < 2. This may be a consequence of the relaxation approach used. Furthermore, in an embedded study where an interview of eight participants was performed [46], most of them indicated that they felt that relaxation had helped improve their motor function and life activities. These data implied that relaxation may play an important role in tai chi practice. Future studies are needed to better understand the role that muscle relaxation plays.

Shoulder pain was evaluated as one of the potential influencing factors for acceptability. The result showed that the shoulder pain of four participants did not interfere with their tai chi movements during sessions and their practice at home. Moreover, the tai chi training did not cause any shoulder pain in the participants without initial shoulder pain. Therefore, shoulder pain does not seem to hinder tai chi practice. Though the initial shoulder pain of four participants decreased after the intervention, the sample size was too small to perform the inferential statistical analysis. Thus, there was insufficient evidence to confirm that reduced shoulder pain was mainly due to the tai chi training. Future studies with larger sample sizes are needed.

The main limitation of this study is that this was a convenience sample with small sample size. There were no participants in stages 4 and 5 to be able to make a complete portrait of the clinical reasoning for adapting tai chi, and a detailed balance guideline was not provided for choosing lower limb positions. Also, the division of subgroups was limited by the small sample size. Future studies with a large sample size may take more situations into account, such as coordination and learning ability, to provide a more complete clinical reasoning picture.

## 5. Conclusions

This exploratory study suggests that adapted tai chi may be feasible and acceptable for upper limb rehabilitation by stroke survivors with different impairment levels with respect to the paretic upper limb and balance. A clinical reasoning algorithm for adapting tai chi based on relaxation and coordination principles can provide recommendations for clinicians and researchers. Tai chi may be a promising upper limb rehabilitation strategy, given that in this study participants pursued a high amount of self-practice at home. Low upper limb function, insufficient balance, spasticity, and shoulder pain do not appear to hinder tai chi practice. Future research is needed to provide a more complete portrait of the clinical reasoning for adapting tai chi.

## Figures and Tables

**Table 1:** Demographic and clinical characteristics of participants.

Subject	Age	Gender	Dominant-Side Hemiparesis	Stroke Type	Time after Onset (Months)	CIRS-G Severity Index (Max 4)	Technical Aids for Transfer	Initial CMSA (Arm)	Initial CMSA (Hand)	Spasticity (MAS) (Max 4)	BTX Injection	Initial Shoulder Pain (VAS) (Max 10)
1	41	M	No	Hem	50	3.0	Cane	2	2	4	Yes	3
2	62	M	No	Isc	24	0	Cane	2	2	3	Yes	3
3	53	M	Yes	Isc	14	0	Cane	2	2	3	Yes	0
4	54	M	Yes	Isc	10	0	Independent	3	2	2	No	0
5	46	M	No	Hem	13	3.3	Cane	3	4	1	No	0
6	65	F	Yes	Isc	56	2.0	Independent	3	2	0	No	0
7	87	M	Yes	Isc	10	4.0	Wheel chair	3	5	1	No	9
8	63	M	No	Hem	41	0	Independent	3	4	2	No	0
9	47	M	No	Isc	8	3.0	Independent	6	6	0	No	7
10	67	M	No	Isc	12	2.5	Independent	6	6	0	No	0
11	68	F	No	Isc	12	3.0	Independent	7	7	0	No	0

Abbreviations: F = female; M = male; Hem = hemorrhagic stroke; Isc = ischemic stroke; CIRS-G, Cumulative Illness Rating Scale for Geriatrics; CMSA, Chedoke-McMaster Stroke Assessments; MAS, Modified Ashworth Scale; BTX = botulinum toxin; VAS = Visual Analogue Scale.
